# Insufficient Efficacy of Corpus Callosotomy for Epileptic Spasms With Biphasic Muscular Contractions

**DOI:** 10.3389/fneur.2020.00232

**Published:** 2020-04-02

**Authors:** Sotaro Kanai, Tohru Okanishi, Mitsuyo Nishimura, Masayoshi Oguri, Hideo Enoki, Yoshihiro Maegaki, Ayataka Fujimoto

**Affiliations:** ^1^Division of Child Neurology, Faculty of Medicine, Institute of Neurological Sciences, Tottori University, Yonago, Japan; ^2^Department of Child Neurology, Seirei-Hamamatsu General Hospital, Hamamatsu, Japan; ^3^Laboratory of Neurophysiology, Seirei-Hamamatsu General Hospital, Hamamatsu, Japan; ^4^Division of Pathobiological Science and Technology, Faculty of Medicine, School of Health Sciences, Tottori University, Yonago, Japan; ^5^Comprehensive Epilepsy Center, Seirei-Hamamatsu General Hospital, Hamamatsu, Japan

**Keywords:** epileptic spasms with biphasic muscular contractions, epileptic spasms, corpus callosum, corpus callosotomy, epilepsy surgery

## Abstract

Corpus callosotomy (CC) is the surgical strategy for drug-resistant epileptic seizures including epileptic spasms (ES). In this study we report a subtype of ES which is accompanied by two consecutive muscular contractions. This subtype has not been previously classified and may emerge via a complex epileptic network. We named these seizures “epileptic spasms with biphasic muscular contractions (ES-BMC)” and analyzed the association between them and CC outcomes. We enrolled 17 patients with ES who underwent CC before 20 years of age, and analyzed the records of long-term video-electroencephalogram (EEG) recordings. The outcomes of CC were ES-free (Engel's classification I) in 7 and residual ES (II to IV) in 10 patients. We statistically analyzed the associations between the presence of preoperative ES-BMC and the outcomes. Ages at CC ranged from 17 to 237 months. We analyzed 4–44 ictal EEGs for each patient. Five patients presented with ES-BMC with 6–40% of their whole ES on the presurgical video-EEG recordings, and all of them exhibited residual ES outcomes following CC. A Fisher's exact test revealed a significant positive correlation between the presence of preoperative ES-BMC and persistence of ES following CC (*p* = 0.044, odds ratio = 15.0, risk ratio = 2.0). The presence of ES-BMC may be useful in the presurgical prediction of CC outcomes in patients with ES.

## Introduction

Epileptic spasms (ES), which are the epileptic seizures commonly associated with West syndrome, are characterized by brief muscle contractions that typically involve the trunk and limbs. ES is associated with typical ictal electroencephalogram (EEG) patterns, including fast waves bursts, high-voltage slow waves (HVSs), and desynchronization (1–3). ES-associated electromyogram (EMG) activity exhibits rhombus patterns, whereby activity quickly reaches a peak and then decreases (1, 2). ES are often medically intractable, and therapeutic approaches, including antiepileptic drugs (AEDs), adrenocorticotropic hormone therapy, and surgical treatments, have been discussed in many studies. However, the treatment strategy for drug-resistant ES has not been integrated.

Corpus callosotomy (CC) is a valuable palliative surgical option for patients with diffuse or multifocal epileptic discharge that results in generalized seizures, especially among those who experience drop attacks (4, 5). The pathophysiologic basis of CC is based on the hypothesis that the corpus callosum is the most important pathway for the spread of epileptic activity between the two hemispheres (6). Some reports have indicated that CC has beneficial effects in patients with ES (7–9). In these studies, ES were eliminated following CC in 42 of 87 patients. The authors presumed that the interruption of corticocortical pathways via the corpus callosum may be critical in the emergence of ES. Furthermore, Ono et al. proposed that interhemispheric recruitment of the epileptogenic state via the corpus callosum accounts for bilateral synchrony, and that disconnection of the transcallosal volleys may lead to seizure elimination in such cases (10).

Although the ictal manifestation of ES is mostly bilateral, it can also be asymmetrical, asynchronous, or focal. Asymmetry, asynchrony, and focal signs always indicate symptomatic etiology (1–3). The authors suggested that ES represent a heterogeneous group of seizures that derive from an age-related cortical-subcortical interaction.

We discovered a novel type of ES in patients with drug-resistant ES, who presented with “epileptic spasms with biphasic muscular contractions (ES-BMC),” which involved two consecutive spastic muscular contractions and ictal polyphasic HVSs. The manifestations of ES-BMC were found to vary greatly among patients, sometimes even in a single patient, and mostly involved unilateral movements, which is suggestive of the complex cortical-subcortical seizure pathways. Therefore, we hypothesized that the corpus callosum might be less involved with the seizure pathway of ES in patients with ES-BMC than in patients without them. In the present study, we retrospectively analyzed the efficacy of CC in patients with ES, and investigated the association between the preoperative presence of ES-BMC and seizure outcomes.

## Materials and Methods

### Patients

Clinical data and video EEG recordings were retrospectively collected from each patient's medical records. The enrolled patients had also participated in another submitted study on the association between ictal HVS symmetry and CC outcomes.

The inclusion criteria were as follows: (1) CC prior to the age of 20 years that was performed between 2008 and 2017 at Seirei-Hamamatsu General Hospital, (2) main seizure type of ES, (3) availability of ictal EEG recordings prior to CC, and (4) a follow-up period after CC > 12 months. Patients with inappropriate EEG recordings (e.g., misplacement of EEG electrodes) or serious surgical complications which can affect seizure prognosis were excluded from the study. Patient information including age, seizure type, etiology, EEG data, and postsurgical seizure outcome was anonymized prior to our analysis. This study was carried out in accordance with the recommendations of the institutional guidelines of Seirei Hamamatsu General Hospital. The protocol was approved by the institutional review boards. All subjects gave written informed consent in accordance with the Declaration of Helsinki.

### Clinical Profiles

For each patient, we reviewed clinical data including sex, age at epilepsy onset, number of AEDs prescribed prior to CC, frequency of ES, classification of epilepsy syndrome or epilepsy type, etiology, age at CC, CC procedure (anterior CC or total CC), and follow-up period. We defined the patients with ES and developmental delay as having generalized or focal epilepsy in accordance with seizure types and the distribution of EEG discharge, based on the 2017 International League Against Epilepsy Classification of the Epilepsies (11).

Seizure outcomes of ES following CC were evaluated based on the degree of remission of ES at the last follow-up, as follows: free (100%), rare (>75%), improved (50–75%), and unchanged (<50%). CC outcomes including all seizure types were evaluated based on the Engel's classification (12).

### Preoperative Scalp Video-EEG Recordings

Scalp video-EEG data were recorded using a NicoletOne or BMSI6000 system (Natus Medical Incorporated, WI, US) for patients 1–4, 6–9, and 11–16, and a Neurofax system (Nihon-Kohden, Tokyo, Japan) for patients 5, 10, and 17. EEG electrodes were placed in accordance with the international 10–20 scalp-electrode position. The sampling rate was set at 256 Hz (patients 3, 8, 12, and 16), 400 Hz (patients 1, 2, 4, 7, 11, 14, and 15), 500 Hz (patients 5, 10, and 17), 512 Hz (patients 6 and 13), or 1,024 Hz (patient 9). Low-cut and high-cut filters were set at 0.5 and 70 Hz for NicoletOne or BMSI6000 and 1.6 and 60 Hz for Neurofax, respectively. EMG electrodes were placed on both deltoid muscles.

### Postoperative Scalp Video-EEG Recordings

We performed scalp video-EEG in 12 patients following CC. We used a NicoletOne system for patients 3, 12, 13, 15, and 16, and a Neurofax system for patients 1, 5, 8, 9, 10, 14, and 17. The sampling rate was set at 256 Hz (patients 3, 12, and 15), 400 Hz (patients 3, 12, 13, and 15), 500 Hz (patients 1, 5, 8, 9, 10, 14, and 17), or 512 Hz (patient 16). For patients 3, 12, and 15, we performed multiple scalp video-EEGs postoperatively. Low-cut and high-cut filters were set at 1 and 40 Hz for NicoletOne, and 1.6 and 60 Hz for Neurofax. The other points were the same as those described in Section Preoperative Scalp Video-EEG Recordings.

### Definitions of ES and ES-BMC

**Figure 1:**
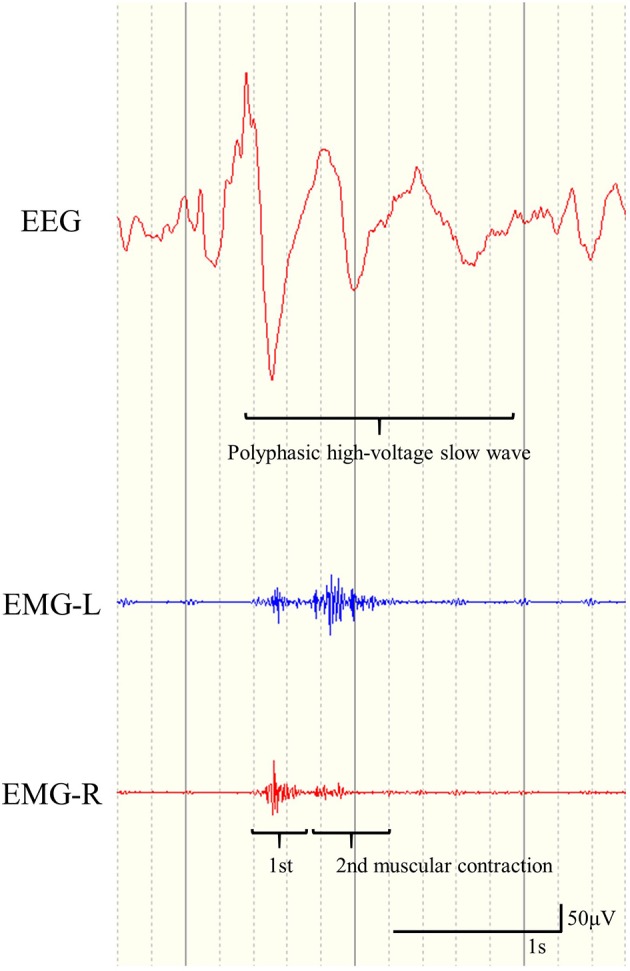
Representative example of electroencephalogram and electromyogram (EMG) findings for epileptic spasms with biphasic muscular contractions. Two sequential rhombus patterns in the EMG recording appear simultaneously with polyphasic high-voltage slow waves.

We defined ES as seizures with brief muscular contractions on EMG with crescendo-decrescendo patterns, and diffuse polyphasic HVSs in ictal EEG that occurred immediately prior to or simultaneously with ictal muscular contractions. Furthermore, we defined ES-BMC as fulfilling all the following characteristics: (1) two sequential spastic muscular contractions that could be recognized visually with video-EEG, (2) two sequential rhombus patterns in ictal EMG, and (3) polyphasic ictal HVSs (Figure 1). We retrospectively analyzed the correlation between the preoperative presence of ES-BMC and CC outcomes. EEG data were analyzed based on the montages of longitudinal bipolar and average reference.

For the four patients for whom we had both preoperative and postoperative video-EEG data containing ES-BMC, we analyzed the transition of seizure semiology, the number of ES and ES-BMC, the difference in the distribution of ictal-HVSs before and after CC, and the latency between two EMG peaks.

### Statistical Analysis

We evaluated whether or not the patients presented ES-BMC in the preoperative video-EEG. We divided patients into two outcome groups, ES-free (Engel's I) or residual ES (II to IV). We analyzed the associations between the presence or absence of preoperative ES-BMC and outcomes of ES-free or residual ES using Fisher's exact test. Analyses were performed using R (version 3.6.0). The level of statistical significance was set at *p* < 0.05.

## Results

### Patient Characteristics

Among 46 patients who underwent CC prior to the age of 20 years, we recruited 17 (15 male and 2 female) who fulfilled the inclusion criteria. One male patient, who developed a brain herniation due to postoperative intracranial hemorrhage, was excluded from the study since this could have affected seizure prognosis. Their clinical profiles are presented in Tables 1, 2. The mean age at epilepsy onset was 23 months (range: 1–166 months), while the mean age at CC was 81.4 months (range: 17–237 months). The age at CC was significantly lower in the residual ES group. Three patients underwent anterior four-fifths CC, while the remaining 14 underwent total CC. The mean follow-up period after CC was 35.2 months (range: 12–75 months). The CC outcomes of ES were free, rare, improved, and unchanged in 7, 2, 4, and 4 patients, respectively; therefore, 7 and 10 patients belonged to the ES-free and residual ES groups, respectively. As per the Engel's classification, the outcomes in 3, 6, 4, and 4 patients were I, II, III, and IV, respectively.

**Table 1:** Clinical information of patients with favorable (free from ES) and unfavorable (others) outcomes.

	**ES free *N* = 7**	**ES residual *N* = 10**	***p*-value**
Sex (boys: girls)	6: 1	9: 1	n.s.
Types of epilepsy syndrome or epilepsy type			n.s.
West syndrome	2	5	
Lennox-Gastaut syndrome	1	2	
CGFE except for Lennox-Gastaut syndrome	4	3	
Etiology			n.s.
Structural abnormality	5	6	
Genetic/chromosomal syndrome	2	1	
Unknown	0	3	
Age at epilepsy onset [months, range (mean)]	4–166 (49)	1–13 (5)	n.s.
Total number of AEDs before CC [range (mean)]	4–8 (6.6)	6–10 (7.3)	n.s.
Frequency of ES			n.s.
1–20/day	5	5	
>20/day	2	5	
Age at CC [months, range (mean)]	45–237 (125)	17–106 (51)	0.042
Procedure of CC			n.s.
Total callosotomy	6	8	
Anterior 4/5 callosotomy	1	2	
Outcomes of Engel's classification			NA
I	3	-	
II	4	2	
III	-	4	
IV	-	4	
Follow-up periods [months, range (mean)]	12–44 (28)	12–75 (40)	n.s.

*ES, epileptic spasms; CGFE, combined generalized and focal epilepsy; AEDs, antiepileptic drugs; CC, corpus callosotomy; n.s., not significant; NA, not applicable. We used Fisher's exact probability test, Welch t-test and chi-square test, appropriately. Tuberous sclerosis complex was classified to structural abnormality in etiology*.

**Table 2:** Clinical Profiles of Each Patient.

**Patient**	**Epilepsy classification**	**Etiology**	**Age at epilepsy onset (months)**	**Preoperative frequency of ES (per day)**	**Preoperative seizure types except ES**	**Age at CC (months)**	**CC procedure**	**CC outcomes of ES**	**Engel's classification**	**Residual seizure types except ES after CC**	**Follow-up period (months)**
1	CGFE	Hippocampal sclerosis	0–6	25	Atonic, FIAS	67–72	Total	Free	II	FIAS	36
2	LGS (CGFE)	FCD	0–6	7	Tonic, atonic, myoclonic	235–240	Total	Free	I	–	24
3	WS	TSC, Acute encephalopathy	0–6	20	Tonic	43–48	Total	Free	I	-	44
4	WS	Acute encephalopathy	7–12	100	–	55–60	Total	Free	I	-	36
5	CGFE	TSC	0–6	20	FIAS	97–102	Total	Free	II	FIAS	28
6	CGFE	Chemotherapy–induced leucoencephalopathy	10–12 (years)	10	Tonic, FIAS	16–18 (years)	Ant. four–fifth	Free	II	FIAS	12
7	CGFE	*MECP2* duplication syndrome, Acute encephalopathy	13–15 (years)	20	FIAS	13–15 (years)	Total	Free	II	FIAS	20
8^*^	WS	Unknown	0–6	50	–	13–18	Total	Rare	II	–	37
9	CGFE	TSC	0–6	5	Tonic, FIAS	61–66	Total	Rare	III	Tonic, FIAS	24
10^*^	CGFE	TSC	0–6	20	Tonic, FIAS	31–36	Total	Improved	II	Tonic, FIAS	12
11^*^	LGS (CGFE)	Neonatal HIE	13–18	100	Tonic	79–84	Ant. four–fifth	Improved	III	Tonic	24
12	WS	VLCAD deficiency	0–6	20	–	37–42	Total	Improved	III	–	75
13	WS	Down syndrome	7–12	10	–	31–36	Total	Improved	III	–	39
14^*^	LGS (CGFE)	Neonatal hypoglycemia	0–6	200	Tonic	43–48	Total	Unchanged	IV	Tonic	33
15^*^	WS	Unknown	7–12	7	Tonic	103–108	Total	Unchanged	IV	Tonic	58
16	CGFE	Unknown	0–6	50	Tonic, FIAS	61–66	Ant. four–fifth	Unchanged	IV	Tonic, FIAS	72
17	WS	TSC	0–6	30	–	25–30	Total	Unchanged	IV	-	25

* *The patient who presented with epileptic spasms with biphasic muscular contractions preoperatively. For patients 6 and 8, the ages are listed in years. CC outcomes for ES were defined as follows. Free: complete cessation of ES, rare: > 75% reduction, improved: 50–75% reduction, and unchanged: <50% reduction. Overall seizure outcome was evaluated based on the Engel's classification. Engel's class I: complete cessation of seizures, class II: > 75% reduction, class III: 50–75% reduction, and class IV: <50% reduction. M, male; F, female; AED, anti-epileptic drug; ES, epileptic spasm; CC, corpus callosotomy; CGFE, combined generalized and focal epilepsy; LGS, Lennox-Gastaut syndrome; WS, West syndrome; FCD, focal cortical dysplasia; TSC, tuberous sclerosis complex; MECP2, methyl-CpG binding protein 2; HIE, hypoxic-ischemic encephalopathy; VLCAD, very-long-chain acyl-CoA dehydrogenase; FIAS, focal onset impaired awareness seizure*.

Seven and 10 patients were diagnosed with West syndrome and combined generalized and focal epilepsy (CGFE), respectively; this included three with Lennox-Gastaut syndrome. While patients 4, 8, 12, 13, and 17 developed only ES, others experienced more than two types of seizures, including tonic seizures, focal onset impaired awareness seizures, atonic seizures, and myoclonic seizures. Patient 3 presented tonic seizures, atonic seizures, and myoclonic seizures preoperatively, all of which were resolved following CC. Tonic seizures were resolved postoperatively in patients in the favorable outcome group, but not in the others. Patients with preoperative focal onset seizures all experienced such seizures following CC. The mean frequency of ES was 40.1 times per day (range: 5–200).

The following etiologies were identified in 16 patients: tuberous sclerosis complex (*n* = 5), post-acute encephalopathy (*n* = 3), hippocampal sclerosis (*n* = 1), focal cortical dysplasia (*n* = 1), chemotherapy-induced leukoencephalopathy (*n* = 1), methyl-CpG binding protein 2 (*MECP2*) duplication syndrome (*n* = 1), post-neonatal hypoxic-ischemic encephalopathy (*n* = 1), very-long-chain acyl-CoA dehydrogenase deficiency (*n* = 1), Down syndrome (*n* = 1), and post-neonatal hypoglycemia (*n* = 1). Patient 3 had both tuberous sclerosis complex and acute encephalopathy, while patient 7 had both *MECP2* duplication syndrome and acute encephalopathy. Three patients had no neurologic history prior to the onset of epilepsy.

### Summary of ES and ES-BMC

**Figure 2:**
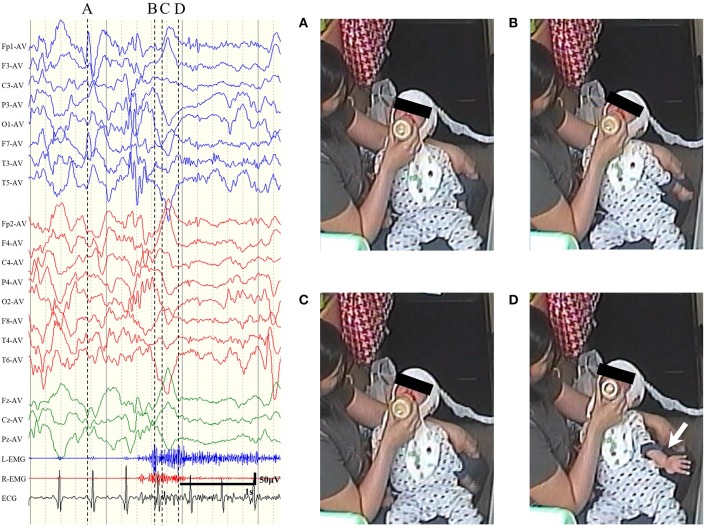
Representative examples of sequential seizure manifestation for epileptic spasms with biphasic muscular contractions of patient 8, pointed out by the dotted line. **(A)** Prior to the seizure. **(B)** At the first peak of the electromyogram (EMG). The neck and the trunk extended. **(C)** At the interval between two EMG peaks. The trunk looked slightly relaxed. **(D)** At the second peak of EMG. The left arm spastically extended out (white arrow).

Preoperative EEG data were reviewed for 216 ES cases. ES in clusters were reviewed individually, and those with unclear videos (e.g., the patient was hidden under the bedclothes or the whole body was not within the filming coverage) and electrographic seizures were excluded. For each patient, we reviewed 4–44 ictal EEGs (mean: 12.7). In accordance with the definitions described in Section Definitions of ES and ES-BMC, we differentiated ES-BMC from ES by carefully assessing ictal video-EEG (Figure 2, see Supplementary Video 1 to refer to the actual video-EEG). There were no patients with clearly focal ES eligible for focal resective surgery. Patient 1 underwent a right anterior temporal lobectomy for focal onset impaired awareness seizures prior to CC. Five patients presented with ES-BMC preoperatively (range: 1–8 times; mean: 4.5), and the Engel's classification following CC were II in 1 patient, III in 2 patients, and IV in 2 patients. No patients with an ES-free outcome (Engel's class I) presented with ES-BMC prior to CC.

**Figure 3:**
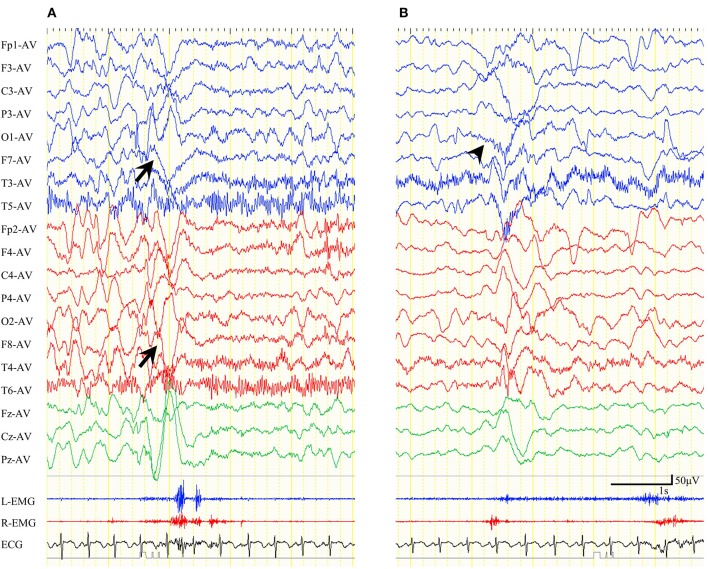
Ictal electroencephalogram (EEG) findings for epileptic spasms with biphasic muscular contractions of patient 10 in an average reference montage. **(A)** Preoperative ictal EEG. Ictal high-voltage slow waves (HVSs) emerged in the bilateral anterior-temporal area. Biphasic electromyogram (EMG) emerged bilaterally. **(B)** Postoperative ictal EEG. Ictal HVSs emerged mainly in the left occipital area (wedge). EMG initially emerged on the right side, and subsequently on the left.

Among five patients with preoperative ES-BMC, we performed video-EEG in four of them between 1 week and 24 months after CC (mean: 7 months). ES-BMC remained in all four patients. The last remaining patient did not perform postoperative video-EEG, since she was referred from another hospital. Other patients did not exhibit ES-BMC in postoperative EEG recordings. In total, 22 ES-BMC were recorded preoperatively (19% of total ES) and 55 were recorded postoperatively (25% of total ES, Table 3). Seizure semiology and ictal HVS distribution were different before and after CC in all four patients. Patient 15 presented with multiple seizure semiology following CC. The seizure manifestations of ES-BMC were entirely asymmetric; namely, the amplitude ratio between bilateral EMG was more than double and/or they presented with unilateral limb movements, both before and after CC. Preoperative ictal HVSs emerged bilaterally but were occasionally asymmetrical, whereas postoperative ictal HVSs were almost always unilateral (Figure 3). The mean latency between the two peaks of the ictal EMG was 432 ms (range: 160–860 ms) preoperatively, and 483 ms (range: 70–1,000 ms) postoperatively.

**Table 3:** Characteristics of epileptic spasms with biphasic muscular contractions (ES-BMC).

**Patient**	**Pre/post CC**	**Number of ES/ ES-BMC**	**Seizure semiology of ES-BMC**			**Ictal HVSs distribution**	**The mean latency between two EMG peaks, ms (range)**
			**1st phase**	**2nd phase**	**Laterality of 1st/2nd phases**		
8	Pre	6/4	Extensor of neck and trunk	Extensor of left arm	Bilateral -> Left	Bilateral pT	290 (270–320)
	Post	0/6	Extensor of trunk	Extensor of right leg	Bilateral –> Right	Left pT	440 (400–470)
10	Pre	39/5	Flexor of trunk and left leg	Flexor of left leg	Left -> Left	Bilateral aT	440 (360–540)
	Post	110/40	Flexor of right arm	Flexor of left leg	Right -> Left	Left O	180 (70–360)
11	Pre	16/4	Flexor of trunk and both arms	Flexor of left shoulder	Bilateral -> Left	Left F	300 (230–360)
	Post	NA	-	-	-	-	–
14	Pre	19/8	Flexor of trunk and left leg	Flexor of left leg	Left -> Left	Bilateral O	270 (160–350)
	Post	24/2	Flexor of both arms	Flexor of both legs	Bilateral -> Bilateral	Right O	580 (290–870)
15	Pre	15/1	Extensor of trunk	Extensor of right arm	Bilateral -> Right	Bilateral Fp	860 (–)
	Post	31/7	Head deviation to the left Extensor of right arm	Extensor of right arm	Left or Right -> Right	Right aT	730 (450–1000)

*CC, corpus callosotomy; ES, epileptic spasms; ES-BMC, epileptic spasms with biphasic muscular contractions; HVSs, high-voltage slow waves; EMG, electromyogram; NA, not available; pT, posterior-temporal; aT, anterior-temporal; O, occipital; Fp, frontopolar*.

### Correlation Results

Fisher's exact test revealed a significant positive correlation between the presence of preoperative ES-BMC and persistence of ES following CC (*p* = 0.044). ES tended to remain following CC in patients with preoperative ES-BMC compared with the patients without them (odds ratio = 15.0, risk ratio = 2.0, Table 4).

**Table 4:** Statistical analyses.

**CC outcomes of ES free or residual**	**Preoperative ES-BMC**		***P*-value**	**Odds ratio**	**Risk ratio**
	**Absence**	**Presence**			
Free	7	0	0.044*	15.0	2.0
Residual	5	5			

For the statistical analyses, we used Fisher's exact test. *P < 0.05. CC, corpus callosotomy; ES, epileptic spasms; ES-BMC, epileptic spasms with biphasic muscular contractions.

## Discussion

### Summary of Findings

In this retrospective study, we investigated the correlation between ES-BMC, which were defined as ES with biphasic spastic muscular contractions and polyphasic ictal HVSs, and CC outcomes. The preoperative presence of ES-BMC was significantly correlated with residual ES following CC. Our findings suggest that the presence of ES-BMC can be used to predict CC outcomes in patients with ES.

### Mechanism of ES Emergence and the Role of the Corpus Callosum

The pathological mechanisms underlying ES remain largely unknown. Previous research has indicated that both the cortex and subcortical structures, including the brainstem, thalamus, and basal ganglia, play a role in the emergence of ES (13, 14). Some researchers have hypothesized that ES are derived from cortical-subcortical interactions, whereby the cortex triggers the activation of subcortical structures, which leads to the generation of ES (2, 14).

Avanzini et al. reported that interhemispheric coherence of fast activity was low prior to ES and that such activity increases following ES, which is indicative of signal transfer through the corpus callosum during the sequential ES state (13). Ono et al. proposed greater involvement of the corpus callosum, and suggested that an interhemispheric recruitment via the corpus callosum precedes cortical spike discharges, which maintains cortical epileptogenic susceptibility in both hemispheres (10). The functional role of the corpus callosum in mutually intensifying epileptogenesis between the bilateral hemispheres in ES may be more prominent in patients who respond favorably to CC.

### Symmetry of ES and the Corpus Callosum

Previous studies have indicated that CC exerts a beneficial effect on approximately half of patients with ES (7–9). The disconnection of the corticocortical pathway via the corpus callosum is thought to eliminate the interhemispheric mutual irritating of ES. Conversely, other authors have reported that CC may be ineffective for reducing ES (15, 16), which suggests that some forms of ES are less affected by the corpus callosum pathway.

The ictal manifestations of ES are mostly symmetrical. Asymmetry, asynchrony, and focal signs of ES always suggest a symptomatic etiology (1–3). Some authors have defined asynchronous spasms as those involving one side of the body before another (3, 17). Although ES-BMC and asynchronous spasms may share some characteristics, ES-BMC mostly involved bilateral synchronous, and sometimes asymmetric, muscular contractions, or two consecutive contractions on one side of the body. Since CC may be ineffective for patients with ES-BMC, our classification may be useful when selecting patients indicated for CC.

In one case report, a patient with West syndrome whose ES were initially bilateral and synchronous developed bilaterally independent ES that originated from either hemisphere after total CC (18). Furthermore, patients with Aicardi syndrome, which is characterized by total or partial agenesis of the corpus callosum and early-onset ES, are reported to present asymmetry in both seizure semiology and ictal HVS of ES (19). These reports indicate that the corpus callosum might account for the symmetry of ES. ES-BMC in the present study mostly exhibited unilateral seizure semiology, particularly in the 2nd phase of the muscular contractions. Therefore, we hypothesized that CC would not resolve ES in patients with ES-BMC.

### A Possible Mechanism Underlying the Emergence of ES-BMC

In this study, of the five patients who presented with both ES and ES-BMC preoperatively, three patients also presented with both ES and ES-BMC after CC, one presented with only ES-BMC, and one did not undergo postoperative video-EEG. The ictal manifestations of ES-BMC were different between the 1st and 2nd phases in three patients, which suggests that they had multiple or complex seizure pathways. One potential mechanism underlying ES-BMC might be the asynchronous epileptic excitations among the bilateral hemispheres, regardless of whether the epileptogenesis is focal or not. Considering that CC did not resolve ES in patients with ES-BMC, the corpus callosum might be less involved in their seizure pathways than the connectivity between cortex, subcortical structure, and brain stem. On the contrary, in the patients with favorable outcomes, the corpus callosum might play a role in forming symmetrical epileptic excitations in the emergence of ES.

### Future Clinical Applications

The epileptogenic cortices of ES are suggested to be widespread (20). Although extensive resective surgeries, including hemispherectomy, and multilobar resection, have been reported to be effective for ES (21, 22), they sometimes give rise to serious complications or sequelae. CC is a less-invasive strategy than such resective surgeries. However, seizure outcomes following CC for ES vary greatly among patients. Accordingly, it is essential to identify factors that allow us to presurgically predict the efficacy of CC.

### Limitations

The present study has some limitations that should be noted. Since there was an insufficient number of patients to perform any multivariate analyses in this study, we could not evaluate the effect of age at CC on the presence of preoperative ES-BMC or identify the best prognostic factors. We assessed the asymmetry of seizure manifestations based on visual analyses of EMG and the video recordings. Since the visual analyses might result in a bias of examiners' subjectivity, a more objective evaluation method is required. The patterns of EEG during the seizures were variable. Therefore, a larger number of patients are necessary to validate the correlations between the EEG findings and ES-BMC, in comparison with ES.

## Conclusions

We hypothesized that favorable outcomes following CC would be associated with involvement of the corpus callosum in the generation of ES. Seizure semiology of ES-BMC primarily involved unilateral movement, which is suggestive of negligible involvement of the corpus callosum. Thus, we focused on the presurgical presence of ES-BMC, which were presumed to respond unfavorably to CC. We found that the presurgical presence of ES-BMC was correlated with poor CC outcomes. Our results indicate that patients with presurgical ES-BMC are predicted to have a poor prognosis following CC, and they should avoid CC and receive other treatments, such as resective surgery, dietary therapy, or vagus nerve stimulation.

## Data Availability Statement

All datasets generated for this study are included in the article/Supplementary Material.

## Ethics Statement

The studies involving human participants were reviewed and approved by Seirei Hamamatsu General Hospital Clinical Research Review Committee. Written informed consent to participate in this study was provided by the participants' legal guardian/next of kin.

## Author Contributions

SK and TO designed the study and performed statistical analyses. SK, MN, and MO acquired and analyzed the EEG data. SK managed the EEG data and wrote the manuscript. HE, AF, and YM critically revised the manuscript. TO and YM discussed the interpretations of the data and revised the manuscript.

### Conflict of Interest

The authors declare that the research was conducted in the absence of any commercial or financial relationships that could be construed as a potential conflict of interest.
